# Dose–Risk and Duration–Risk Relationships between Aspirin and Colorectal Cancer: A Meta-Analysis of Published Cohort Studies

**DOI:** 10.1371/journal.pone.0057578

**Published:** 2013-02-25

**Authors:** Xiaohua Ye, Jinjian Fu, Yi Yang, Sidong Chen

**Affiliations:** 1 School of Public Health and Tropical Medicine, Southern Medical University, Guangzhou, Guangdong, China; 2 School of Public Health, Guangdong Key Laboratory of Molecular Epidemiology, Guangdong Pharmaceutical University, Guangzhou, Guangdong, China; 3 Liuzhou Municipal Maternity and Child Healthcare Hospital, Liuzhou, Guangxi, China; Vanderbilt University, United States of America

## Abstract

**Background:**

In previous meta-analyses, aspirin use has been associated with reduced risk of colorectal cancer. However, uncertainty remains on the exact dose–risk and duration–risk relationships.

**Methods:**

We identified studies by searching several English and Chinese electronic databases and reviewing relevant articles. The dose-response meta-analysis was performed by linear trend regression and restricted cubic spline regression. Subgroup analyses were conducted to explore possible heterogeneity among studies. Potential heterogeneity was calculated as *Q* statistic and *I* ^2^ value. Publication bias was evaluated using funnel plots and quantified by the Begg’s and Egger’s test.

**Results:**

Twelve studies were included in this meta-analysis. An inverse association between aspirin use and colorectal cancer was observed in both the overall group (RR = 0.74, 95% CI 0.64–0.83 for aspirin dose; RR = 0.80, 95% CI 0.75–0.85 for frequency of aspirin use; RR = 0.75, 95% CI 0.68–0.81 for years of aspirin use) and subgroups stratified by sex and cancer site. The dose-response meta-analysis showed that there was a 20% statistically significant decreased risk of colorectal cancer for 325 mg aspirin per day increment, 18% decreased risk for 7 times aspirin per week increment and 18% decreased risk for 10 years aspirin increment.

**Conclusion:**

Long-term (>5 years), low-dose (75–325 mg per day) and regular aspirin use (2–7 times per week) can effectively reduce the risk of colorectal cancer.

## Introduction

Colorectal cancer (CRC) is the third most common cancer and the fourth leading cause of cancer-related deaths worldwide [1], thus effective chemoprevention agents would have important implications for public health. Aspirin use has been associated with reduced risk of CRC in both case-control and cohort studies [2]–[6]. The mechanism of protection has been attributed to the inhibition of a cyclooxygenase (COX)-2 pathway to decrease cellular proliferation, inhibit angiogenesis, and induce apoptosis [7], [8]. Some studies suggest other possible biological mechanisms of action for aspirin that do not involve the COX-2 pathway, but involve the insulin-related pathway, the inhibition of nuclear factor-kappaβ, or the up-regulation of tumor suppression genes [9]–[11].

Two meta-analyses of epidemiological studies on the association between aspirin and CRC separately reported 29% [12] and 27% [13] reduction in the risk of CRC with aspirin use. However, the dose–risk and duration–risk relationships are not clear. A few cohort studies reported an inverse dose-response association between aspirin use and CRC incidence [5]–[7], while other cohort studies found no association or an elevated association between the two [14]–[16]. Therefore, we sought to address the unresolved issue of whether there is a dose-response relationship between aspirin use and CRC risk, and explore the optimal dose and duration of aspirin use in prevention of CRC, so as to guide rational use of aspirin as a chemopreventive agent against cancers.

## Materials and Methods

### Literature Search

The meta-analysis was conducted following the PRISMA guidelines [17] and the PRISMA checklist was listed in Table S1. A systematic search of MEDLINE, PUBMED, EMBASE, ISI WEB, Web of Science, WANFANG and CNKI was performed to identify epidemiological studies published from January 1990 to June 2012. The search was conducted using any combination of the keywords: [aspirin or NSAID or ‘nonsteroidal anti-inflammatory drugs’] and [‘colorectal neoplasms’ or ‘CRC’ or ‘colorectal carcinoma’] and risk. In addition, the content pages of the major epidemiological journals and the reference lists of relevant and review articles were reviewed manually. The search was limited to human studies and English language was used.

### Inclusion and Exclusion Criteria

The inclusion criteria required studies to: 1) have a cohort study design; 2) provide information on aspirin use in relation to CRC; 3) include three or more quantitatively measured exposure categories of aspirin use (such as dose, frequency and duration); 4) have CRC incidence as defined above as the endpoint; and 5) report original data and include hazard ratios (HR) or relative risks (RR) and their 95% confidence intervals (CIs) (or information allowing us to compute them). Studies were excluded if: 1) they were not published as full reports, such as conference abstracts and letters to editors; 2) a cross-sectional or case-control design was used; 3) they were based on selected patients with specific diseases (such as adenomas, ulcerative colitis or prior cancer). When multiple reports were published on the same population or subpopulation, we selected in the meta-analysis only the most recent and informative one.

### Data Extraction

Data extracted from each study included the name of the first author, publication year, country, age, sex, number of subjects, follow-up years, site of cancer, adjustments, outcome measures, aspirin exposure categories (including dose, frequency and duration), RRs of CRC and corresponding 95% CIs for each category of aspirin use. Throughout this paper, RR is used to refer to all risk estimates including RRs or HRs. Two investigators (XH Ye and JJ Fu) independently reviewed and cross-checked the data, and disagreements were resolved by consensus.

### Rescaling of Exposure

When intervals of aspirin categories were reported, the midpoint of the interval was chosen. For the open-ended upper interval, there are three methods to derive the midpoint: 1) 20% higher than the low end of the interval [18]; 2) 50% higher than the low end of the interval [19]; 3) 75% of the width of the preceding category’s range was added to the lower bound [20]. We chose the first method (20% higher than the low end of the interval), which proved to produce a better fit than others.

### Statistical Analysis

We first quantified the association of aspirin use with CRC risk using meta-analysis of RR estimates associated with the highest and the lowest category of aspirin intake. Secondly, subgroup analyses were performed according to sex (men or women) and cancer site (colon or rectum) to explore the CRC risk of each subgroup.

To derive the dose-response relationship between aspirin use and risk of CRC, we used the model proposed by Orsini and Greenland [21], [22] to pool the risk estimates. We first used a restricted cubic spline regression model (Stata RC_SPLINE command) with three knots to create spline variables, then derive the generalized least squares trend estimation (Stata GLST command) by including spline variables. We also fitted another linear regression model without the spline terms. Lastly, the significance of any non-linearity was examined by the likelihood ratio test that compared the model with the linear term only and the model with both the linear and the cubic spline terms.

Statistical heterogeneity between studies was examined using both the Cochrane *Q* statistic (significant at *P*<0.1) and the *I* ^2^ value. *I* ^2^ ranges of 25%–50%, 50%–75%, and ≥75% were considered to represent low, moderate, and high heterogeneity respectively [23], [24]. Subgroup analysis was used to explore the influence of sex and cancer site in the heterogeneity.

Publication bias was evaluated by visual inspection of Begg’s funnel plot and tested by the Begg’s test and Egger’s test (significant at *P*<0.1) [25], [26]. In addition, the trim-and-fill method (Stata METATRIM command) was used to adjust the pooled RR and 95% CI if observed publication bias existed [27]. All statistical analyses were completed using Stata statistical software version 10.0.

## Results

### Characteristics of Included Studies

The literature search and study selection process were shown in Figure S1. Twelve studies were included in the present meta-analysis, including five studies on dose of aspirin use [4], [15], [28]–[30], ten studies on frequency of aspirin use [4]– and nine studies on duration of aspirin use [4], [5], [15], [28]–[33]. All studies were based on the cohort design. Among the participants, 18750 incident cases of CRC occurred during follow-up periods ranging from three to twenty-four years. Eight of these studies were conducted in the United States, while four were in Europe [16], [28], [30], [33] (Tables 1, 2, 3).

**Table 1:** Epidemiological studies of aspirin dose (mg/week) and colorectal cancer.

Author, year	Location	Aspirin dose(mg/week)	Dose midpoint(mg/week)	RR	Adjustment
Chan-2008 [4]	USA	0	0	1	Multivariate-adjusted
		162.5–487.5	325	0.94	
		650–1625	1138	0.78	
		1950–4550	3250	0.72	
Chan-2005 [15]	USA	0	0	1	Multivariate-adjusted
		162.5–487.5	325	1.10	
		650–1625		0.89	
		1950–4550	3250	0.78	
		>4550	5460	0.68	
García Rodríguez-2001 [28]	United Kingdom	0	0	1	Age and sex
		525	525	1.1	
		1050	1050	0.98	
		2100	2100	0.60	
Allison-2006 [29]	USA	0	0	1	Multivariate-adjusted
		<2275	1141	1.12	
		≥2275	2730	0.88	
Larsson-2006 [30]	Sweden	0	0	1	Multivariate-adjusted
		500	500	0.83	
		1000–3000	2000	0.88	
		>3000	3600	0.77	

RR, Relative risk.

**Table 2:** Epidemiological studies of Frequency of aspirin use (times/week) and colorectal cancer.

Author, year	Location	Frequency of aspirin use (times/week)	Frequency midpoint(times/week)	RR	Adjustment
Chan-2008 [4]	USA	0	0	1	Multivariate-adjusted
		0.5–1.5	1	0.94	
		2–5	3.5	0.78	
		6–14	10	0.72	
Theodore-2012 [5] (Men)	Washington	0	0	1	Multivariate-adjusted
		<4	2	0.97	
		≥4	4.8	0.55	
Theodore-2012 [5] (Women)	Washington	0	0	1	Multivariate-adjusted
		<4	2	0.53	
		≥4	4.8	0.84	
Ruder-2011 [6]	USA	0	0	1	Multivariate-adjusted
		<1	0.5	0.96	
		1–6	3.5	0.88	
		≥7	8.4	0.86	
Mahipal-2006 [14]	Iowa,USA	0	0	1	Multivariate-adjusted
		≤1	0.5	0.87	
		2–5	3.5	0.79	
		≥6	7.2	0.76	
Chan-2005 [15]	USA	0	0	1	Multivariate-adjusted
		0.5–1.5	1	1.10	
		2–5	3.5	0.89	
		6–14	10	0.78	
		>14	16.8	0.68	
Friis-2009 [16]	Denmark	0	0	1	Multivariate-adjusted
		1–6	3.5	0.87	
		≥7	8.4	0.73	
Allison-2006 [29]	USA	0	0	1	Multivariate-adjusted
		<7	3.5	1.12	
		≥7	8.4	0.88	
Larsson-2006 [30]	Sweden	0	0	1	Multivariate-adjusted
		1	1	0.83	
		2–6	4	0.88	
		>6	7.2	0.77	
Jacobs-2007 [31]	USA	0	0	1	Multivariate-adjusted
		1–6	3.5	0.87	
		≥7	8.4	0.68	

RR, Relative risk.

**Table 3:** Epidemiological studies of years of aspirin use and colorectal cancer.

Author, year	Location	Years of aspirin use	Years midpoint	RR	Adjustment
Chan-2008 [4]	USA	0	0	1	Multivariate-adjusted
		1–5	3	0.86	
		6–10	8	0.78	
		11–15	13	0.73	
		>15	18	0.68	
Theodore -2012 [5] (Men)	Washington	0	0	1	Multivariate-adjusted
		<4	2	0.97	
		≥4	4.8	0.55	
Theodore -2012 [5] (Women)	Washington	0	0	1	Multivariate-adjusted
		<4	2	0.53	
		≥4	4.8	0.84	
Chan-2005 [15]	USA	0	0	1	Multivariate-adjusted
		1–5	3	1.04	
		6–10	8	0.89	
		11–20	15.5	0.67	
		>20	24	0.68	
García Rodríguez-2001 [28]	United Kingdom	0	0	1	Age and sex
		<0.5	0.25	1.00	
		≥0.5	0.6	0.90	
Allison-2006 [29]	USA	0	0	1	Multivariate-adjusted
		1–5	3	0.94	
		5.1–10	7.5	1.29	
		10.1–20	15	0.58	
		>20	24	0.77	
Larsson-2006 [30]	Sweden	0	0	1	Multivariate-adjusted
		1–10	5.5	0.96	
		11–20	15.5	0.87	
		>20	24	0.65	
Jacobs-2007 [31]	USA	0	0	1	Multivariate-adjusted
		<5	2.5	0.85	
		≥5	6	0.68	
Giovannucci-1995 [32]	USA	0	0	1	Age
		1–4	2.5	1.06	
		5–9	7	0.84	
		10–19	14.5	0.70	
		≥20	24	0.56	
Vinogradova-2007 [33]	United Kingdom	0	0	1	Multivariate-adjusted
		<1	0.5	1.03	
		1–2	1.5	0.91	
		≥2	2.4	0.88	

RR, Relative risk.

### Highest Versus Lowest Dose, Frequency and Duration of Aspirin Used

**Figure 1:**
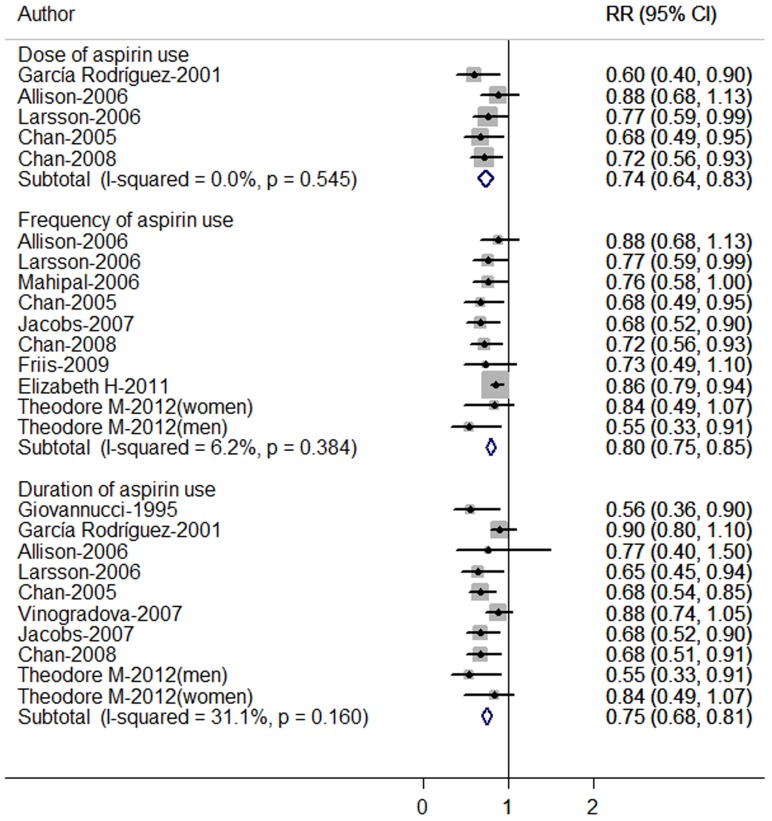
Relative risk of colorectal cancer for highest vs lowest categories of aspirin use (dose, frequency and duration). The combined relative risk was achieved using fixed-effects model. Grey square represents relative risk in each study, with square size reflecting the study-specific weight and the 95% CI represented by horizontal bars. The diamond indicates summary risk estimate.

The overall RRs of CRC comparing categories of the highest and the lowest dose, frequency and duration of aspirin used were presented in Figure 1. Significant reduction in risk of CRC was observed in relation to dose (RR = 0.74, 95% CI 0.64–0.83), frequency (RR = 0.80, 95% CI 0.75–0.85), and duration (RR = 0.75, 95% CI 0.68–0.81) of aspirin used. There was no evidence of heterogeneity among studies on dose (*P* for heterogeneity = 0.545, *I* ^2^ = 0.0%), frequency (*P* for heterogeneity = 0.384, *I* ^2^ = 40.7%), and duration (*P* for heterogeneity = 0.160, *I* ^2^ = 31.1%).

Cancer site (colon or rectum) and sex (men or women) in association of aspirin use were assessed separately (Table 4). The RR estimates from these subgroup analyses varied only slightly and there is no significant difference, indicating that aspirin intake was consistently associated with a decreased risk of CRC. The number of studies on the dose of aspirin use was too small to perform subgroup analysis on.

**Table 4:** Summary of pooled RRs of colorectal cancer by sex and cancer site.

Subgroup	Frequency of aspirin use			Duration of aspirin use		
	Number of studies	Pooled RR (95%CI)	*P*-value*	Number of studies	Pooled RR (95%CI)	*P*-value*
All studies	9	0.80(0.75–0.85)		9	0.75(0.68–0.81)	
Sex						
Men	3	0.60 (0.27–0.93)	0.330	3	0.70(0.54–0.86)	0.767
Women	5	0.82(0.73–0.91)		5	0.73(0.62–0.84)	
Cancer site						
Colon	5	0.76(0.65–0.87)	0.788	2	0.67(0.44–0.91)	0.608
Rectum	5	0.74(0.64–0.83)		2	0.54(0.20–0.88)	

RR, Relative risk;

* represents the heterogeneity of relative risks between subgroups.

**Figure 2:**
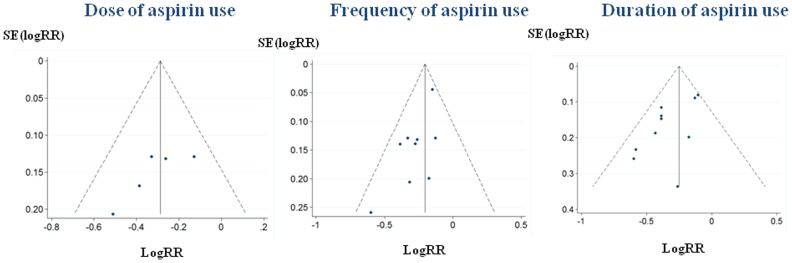
Begg’s funnel plot with 95% confidence limits to detect publication bias. Each point represents a separate study for the indicated association.

Visual inspection of funnel plot and statistical tests suggested no indication of publication bias for studies on dose (Figure 2; Begg’s test *P* = 0.221; Egger’s test *P* = 0.119). Slight publication bias for studies on frequency (Figure 2; Begg’s test *P* = 0.076; Egger’s test *P* = 0.019) and duration (Figure 2; Begg’s test *P* = 0.283; Egger’s test *P* = 0.025) was noted. The RR estimates varied only slightly after using the trim-and-fill method to adjust the potential publication bias (RR = 0.75, 95% CI 0.66–0.85, for dose; RR = 0.82, 95% CI 0.76–0.87, for frequency; RR = 0.75, 95% CI 0.67–0.84, for duration), indicating that aspirin use was consistently associated with a decreased risk of CRC.

### Dose-response Analysis

**Figure 3:**
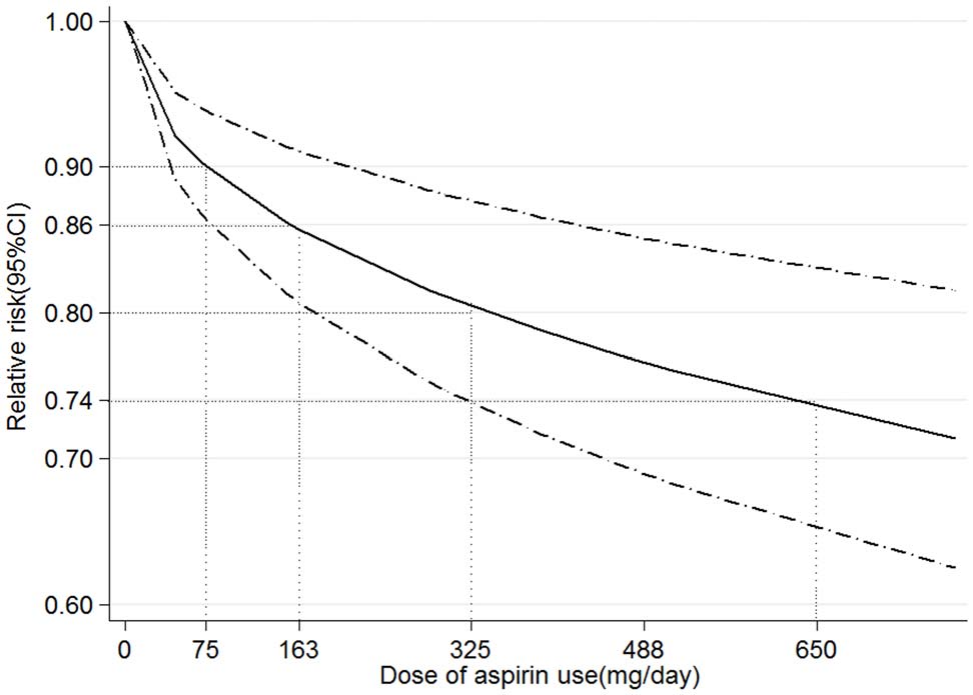
Association between dose of aspirin use and risk of colorectal cancer obtained by spline regression with 3 knots (0, 163, 488 mg per day) and 0 mg per day as reference. *P*_non-linearity_ = 0.020. Solid line represents the estimated relative risk and the dot-dashed lines represent the 95% confidence intervals. The dotted lines are used to explain the relative risk of colorectal cancer for different dose of aspirin use (RR = 0.90, 95% CI 0.86–0.94, for 75 mg per day; RR = 0.86, 95% CI 0.81–0.91, for 163 mg per day; RR = 0.80, 95% CI 0.74–0.88, for 325 mg per day; RR = 0.74, 95% CI 0.65–0.83, for 650 mg per day).

In a random effects cubic spline model that included all studies on dose of aspirin use (mg/day), we found evidence suggesting a non-linear relation between dose of aspirin use and CRC risk (*P* for non-linearity = 0.020; Figure 3). The decreased risk of CRC for 75 mg per day increment of aspirin was 0.90 (95% CI 0.86–0.94), and there was stronger risk reduction for higher aspirin dose (RR = 0.80, 95% CI 0.74–0.88, for 325 mg per day and RR = 0.74, 95% CI 0.65–0.83, for 650 mg per day ).

**Figure 4:**
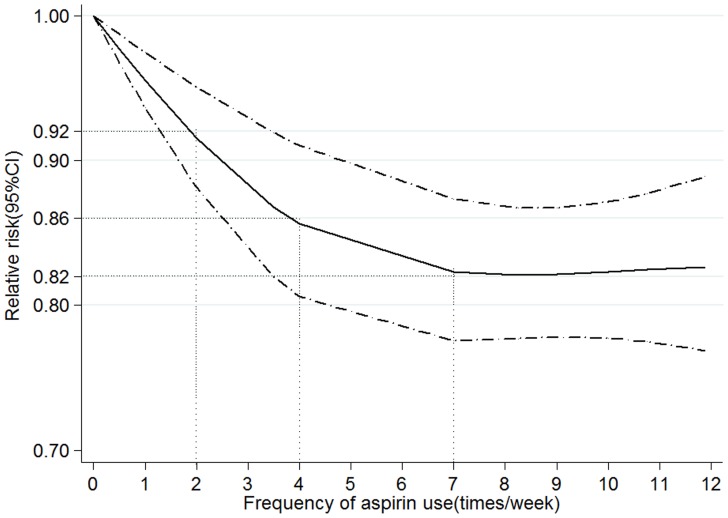
Association between frequency of aspirin use and risk of colorectal cancer obtained by spline regression with 3 knots (0, 3.5, 10.5 times per week) and 0 times per week as reference. *P*_non-linearity_ = 0.007. Solid line represents the estimated relative risk and the dot-dashed lines represent the 95% confidence intervals. The dotted lines are used to explain the relative risk of colorectal cancer for different frequency of aspirin use (RR = 0.92, 95% CI 0.88–0.95, for twice per week; RR = 0.86, 95% CI 0.81–0.91, for 4 times per week; RR = 0.82, 95% CI 0.78–0.87, for 7 times per week; RR = 0.82, 95% CI 0.78–0.87, for 10 times per week).

The random effects cubic spline model that included all studies on frequency of aspirin use (times/week) indicated a non-linear relation between CRC risk and frequency of aspirin use (*P* for non-linearity = 0.007; Figure 4). The decreased risk of CRC for twice per week aspirin user was 0.92 (95% CI 0.88–0.95), and there was a stronger risk reduction for 7 times per week aspirin user (RR = 0.82, 95% CI 0.78–0.87). However, there wasn’t a stronger risk reduction for more than 7 times per week increment (RR = 0.82, 95% CI 0.78–0.87, for 10 times per week).

**Figure 5:**
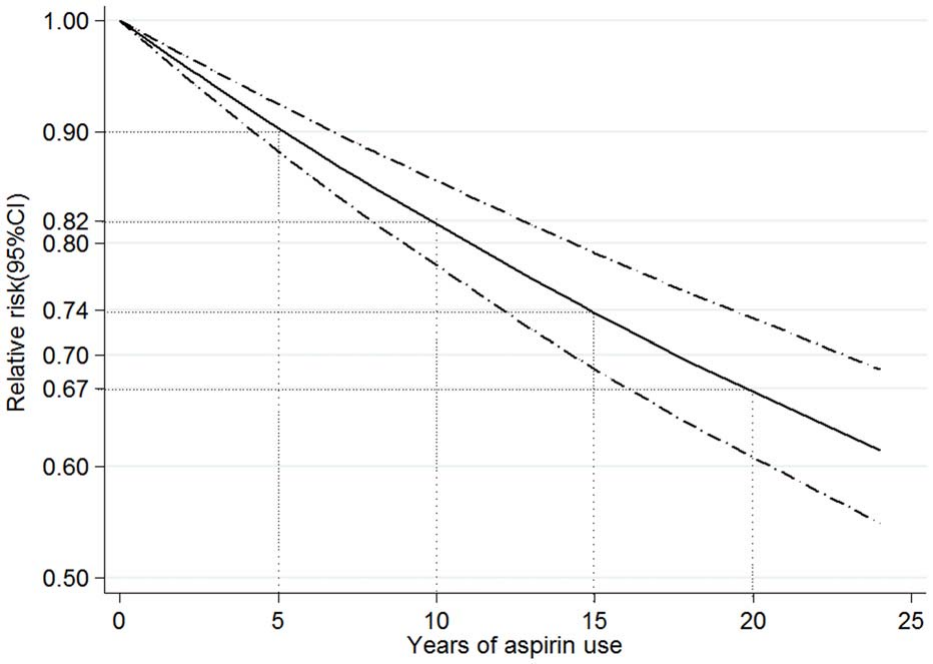
Association between years of aspirin use and risk of colorectal cancer obtained by linear dose-response meta-analyses. *P*_linearity_ = 0.000. Solid line represents the estimated relative risk and the dot-dashed lines represent the 95% confidence intervals. The dotted lines are used to explain the relative risk of colorectal cancer for different duration of aspirin use (RR = 0.90, 95% CI 0.88–0.92, for 5 years of aspirin use; RR = 0.82, 95% CI 0.78–0.86, for 10 years; RR = 0.74, 95% CI 0.69–0.79, for 15 years; RR = 0.67, 95% CI 0.61–0.73, for 20 years).

The non-linear relation between CRC risk and duration of aspirin use had no significance in the cubic spline model (*P* for non-linearity = 0.187), so a linear regression model was fitted (*P* for linear trend<0.001; Figure 5). The risk of CRC declined progressively as the duration of aspirin use increased. The risk of CRC for 5 years of aspirin use was 0.90 (95% CI 0.88–0.92). There was a tendency of stronger risk reduction for longer aspirin used (RR = 0.82, 95% CI 0.78–0.86, for 10 years and RR = 0.67, 95% CI 0.61–0.73, for 20 years).

## Discussion

In this meta-analysis we observe an inverse association between aspirin intake and CRC risk. The CRC risk reduction is around 20%–26%. This finding is consistent with the previous pooled analysis of observational study [13], which reported around 20%–30% reduction in the risk of CRC for regular aspirin use. However, that study included the case-control studies which might subject to selection and recall bias, leading to heterogeneous results. In the present study, we include all the cohort studies published from 1990 to 2012. There is no evidence of heterogeneity among all studies included in this analysis, thus the results are more accurate. Data from randomized clinical trials (RCTs) showed that aspirin consumption not only reduced the risk of colorectal adenomas occurrence or recurrence [34]–[37], but also reduced the incidence, distant metastasis and mortality of CRC [38]–[42]. In conclusion, evidence from RCTs and observational studies supported a beneficial role of aspirin on CRC.

However, previous studies did not provide dose–risk relationship between aspirin intake and CRC risk. In the present study, we performed a meta-analysis of dose-response relationship between aspirin intake and the risk of CRC. Using data on frequency of aspirin use, a monotonically decreasing relationship was observed for low-frequency aspirin intake (RR = 0.92 for twice per week aspirin user; RR = 0.82 for 7 times per week), but the risk reduction levels off for high-frequency aspirin intake (RR = 0.82 for >7 times per week). This trend is consistent with the recent pooled analysis of all cancer risk from case-control studies reporting different frequency categories of aspirin use, but the dose–risk relationships between aspirin and CRC risk are unknown in that study [38]. An interesting and novel finding in our study is the existence of a threshold effect between frequency of aspirin use and the risk of CRC. The threshold of aspirin associated with the risk of CRC is daily user, indicating no stronger risk reduction for >daily aspirin users. For data on aspirin dose, it shows a significant dose–risk relationship. One novel finding is that even with low-dose aspirin intake (<75 mg/day), a reduction in CRC risk was observed, although it is less than 10%. Noted that two RCTs of low-dose aspirin did not show any reduction in the risk of colorectal cancer [43], [44], and a pooled analysis of two RCTs of high-dose aspirin indicated that regular use of at least 300 mg aspirin daily is effective in the primary prevention of colorectal cancer [41]. It was noteworthy that there was a 20% CRC risk reduction for 325 mg/day aspirin intake, but only a 26% risk reduction for double dose in our study. This result implicated that the scale of risk reduction is smaller and smaller with increasing dose of aspirin intake. However, it worth debating whether it is cost-effective using high-dose aspirin as chemoprevention against CRC. Given the greater risk of bleeding complications caused by high-dose aspirin [45] and its cost-effectiveness, the optimal dose of aspirin for prevention of CRC may be within the range of 75–325 mg per day and 2–7 times a week, in which monotonically decreasing dose-response relationships and a more than 10% reduction in risk of CRC were observed. There was some evidence that 81–325 mg daily aspirin use can reduce the incidence and recurrence of colorectal adenomas [35]–[37], [46], and that the recommended dose of aspirin for secondary prevention of cardiovascular disease is 75–150 mg daily [47]. The overlapping confidence intervals suggest that the optimal dose recommended in our study can prevent colorectal adenomas and cardiovascular disease simultaneously.

The duration–risk relationship between years of aspirin use and CRC risk is still unclear. We performed a meta-analysis of dose-response relationship using data on years of aspirin use. An interesting finding is that a negative linear correlation between CRC risk and duration of aspirin use was observed. The risk of CRC decreased by 10% for 5 years increment of aspirin use, and the decreased risk is almost double when aspirin use continued for 10 years. Three meta-analyses from randomized and observational studies [13], [38], [41] also reported that there was a stronger risk reduction for longer aspirin use, and the beneficial effect of aspirin on CRC was evident for those using aspirin for more than 5 years. Therefore, long-term (at least 5 years) aspirin use is recommended in prevention of CRC.

In subgroup analyses by cancer site, there is no difference in the apparent effect on colon caner and rectal cancer. This finding is consistent with the previous pooled analysis [13]. However, five randomized trials did report that benefit of aspirin was greater for cancers in proximal colon than that in distal colon cancers [40]. But studies on proximal and distal colon cancers are too limited to perform subgroup analysis on. Given the limited number of cases within each subgroup, these findings should be interpreted with caution. Therefore further studies should explore the potential different effect of aspirin use based on cancer site, which may relate to the difference in normal physiology, risk factors, mechanisms of carcinogenesis, and the molecular and genetic characteristics of the cancers [48]–[51].

Although the biological mechanisms are uncertain, there is some evidence that the protective effect of aspirin on cardiovascular disease may differ by sex, based on meta-analyses of the randomized trials [52], [53]. However, there is no difference in sex for aspirin use and CRC risk in our study. This finding is consistent with the previous evidence from randomized and observational studies [41]. Because of the limited number of cases within each subgroup, caution should be taken in interpreting results. Confirmatory studies are needed to explore the potential differences in sex of the protective effect of aspirin.

Several potential limitations of this meta-analysis are worth discussing. Firstly, the inherent limitation of observational studies on aspirin use that is related in particular to measurement errors in the exposure to aspirin and the variability of aspirin use definition across studies. These inconsistencies may partly explain the heterogeneity in risk estimates across studies. Secondly, although we included only the results from the fully adjusted models, the results may still be subjected to residual confounding or other biases because different studies may have adjusted for different covariates. Thirdly, we did not attempt to uncover unpublished studies and only included studies which had three or more quantitatively measured exposure categories of aspirin use (such as dose, frequency and duration), which could bring a publication bias and the effect of aspirin as a chemopreventive may be over emphasized. However, visual inspection of funnel plot and statistical tests suggest no indication of publication bias for studies on dose, and only slight publication biases for studies on frequency and duration are noted. Moreover, the RR estimates vary only slightly after using the trim-and-fill method to adjust the potential publication bias. Lastly, both dose and duration of aspirin use have been shown to influence the risk of CRC, but most of the included studies did not provide data on cumulative dose (tablet-years). Consequently, we did not have sufficient data to evaluate the risk of CRC associated with cumulative dose.

The strengths of this meta-analysis are as follows. The aspirin data were observed prospectively, thus minimizing the influence of errors in recall and biases related to incomplete data collection from participants, especially those with fatal diagnoses. In addition, there is no evidence of heterogeneity among all studies included in this analysis. Moreover, the information on the relation with dose, frequency, and duration of aspirin use was considered in order to better understand the dose–risk and duration–risk relationships.

In conclusion, a completely novel finding in our study is the existence of a threshold effect between aspirin intake and the risk of CRC, suggesting that the recommended dose of aspirin for prevention of CRC is 75–325 mg daily and 2–7 times per week. In addition, linear dose-response relationship was observed between duration of use and cancer protection, so long-term (>5 years) consistent use of aspirin appears necessary to achieve a protective effect. In conclusion, long-term, low-dose and regular aspirin use is associated with a reduced risk of CRC. The potential harms associated with aspirin use and the cost-effectiveness in certain high-risk groups must be considered before translating these results into clinical practice.

## Supporting Information

Figure S1 PRISMA Flow Diagram of literature search and study selection.
(DOC)

Table S1 PRISMA checklist of this meta-analyse.
(DOC)
