# Supplementary material for: Dose–Risk and Duration–Risk Relationships between Aspirin and Colorectal Cancer: A Meta-Analysis of Published Cohort Studies
Source: PLoS One. 2013 Feb 25;8(2):e57578. doi: 10.1371/journal.pone.0057578 (PMC3581483; doi:10.1371/journal.pone.0057578)
Supplement: Figure S1 — PRISMA Flow Diagram of literature search and study selection. (DOC) [file pone.0057578.s001.doc]

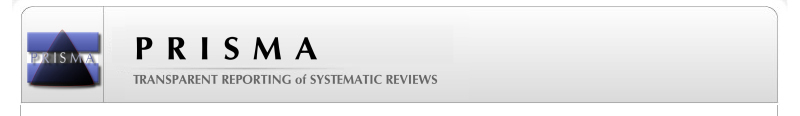
**PRISMA Flow Diagram**

**Screening**

**Included**

**Eligibility**

**Identification**

Records identified through database searching
(n =3340 )

Additional records identified through other sources
(n =6 )

Records after duplicates removed
(n = 2910 )

Records screened
(n =2910 )

Records excluded
(n =2881 )

Full-text articles assessed for eligibility
(n = 29 )

Full-text articles excluded, with reasons
(n =17 )

Studies included in qualitative synthesis
(n =12 )

Studies included in quantitative synthesis (meta-analysis)
(n = 12 )
